# Antimicrobial Activity of* Croton macrostachyus* Stem Bark Extracts against Several Human Pathogenic Bacteria

**DOI:** 10.1155/2016/1453428

**Published:** 2016-05-11

**Authors:** Jackie K. Obey, Atte von Wright, Jimmy Orjala, Jussi Kauhanen, Carina Tikkanen-Kaukanen

**Affiliations:** ^1^Institute of Public Health and Clinical Nutrition, University of Eastern Finland, Kuopio Campus, 70211 Kuopio, Finland; ^2^Department of Medical Laboratory Sciences, School of Health Sciences, University of Eastern Africa, Baraton, Eldoret 30100, Kenya; ^3^Department of Medicinal Chemistry and Pharmacognosy, University of Illinois at Chicago, Chicago, IL 60607, USA; ^4^Ruralia Institute, University of Helsinki, 50100 Mikkeli, Finland

## Abstract

In Kenya, leaves and roots from* Croton macrostachyus* are used as a traditional medicine for infectious diseases such as typhoid and measles, but reports on possible antimicrobial activity of stem bark do not exist. In this study, the antibacterial and antifungal effects of methanol, ethyl acetate and butanol extracts, and purified lupeol of* C. macrostachyus* stem bark were determined against important human gram-negative pathogens* Escherichia coli*,* Salmonella typhi*,* Klebsiella pneumoniae*, and* Enterobacter aerogenes*, gram-positive* Listeria monocytogenes,* and a fungus* Candida albicans*. The most promising broad scale antimicrobial activity against all the studied pathogens was shown by the ethyl acetate extract. The ethyl acetate extract induced the zone of inhibition between 10.1 ± 0.6 mm and 16.0 ± 1.2 mm against* S. typhi*,* E. coli*,* K. pneumoniae*,* E. aerogenes,* and* L. monocytogenes* with weaker antimicrobial activity against* C. albicans *(zone of inhibition: 5.6 ± 1.0 mm). The antibiotic controls (amoxicillin, ciprofloxacin, ampicillin, benzylpenicillin, clotrimazole, and cefotaxime) showed antimicrobial activity with zones of inhibition within 13.4 ± 0.7–22.1 ± 0.9 mm. The ethyl acetate extract had MIC in the range of 125–250 mg/mL against all the studied bacteria and against* C. albicans* MIC was 500 mg/mL. The present results give scientific evidence and support the traditional use of* C. macrostachyus* stem bark as a source for antimicrobials. We show that* C. macrostachyus* stem bark lupeol is a promising antimicrobial agent against several important human pathogens.

## 1. Background

About 80% of the rural population in Sub-Saharan Africa depends on traditional herbal remedies for primary health care and veterinary use [1]. In many developing countries antibiotic resistance, adverse drug reactions, and the high costs of antimicrobials have made management of infectious diseases ineffective [2, 3]. Natural products of higher plants may be a source of new antimicrobial agents with possibly novel mechanisms of action [3, 4].

The plant* Croton macrostachyus* has been traditionally used to treat diseases in Kenya [5]. Stem barks and twigs of* Croton *spp. contain fatty acids, lupeol, and betulin [6]; saponins and resins are usually found in the seeds [6] and crotepoxide and crotomacrine in fruits [7]. Roots contain chalcone and secondary metabolites such as 3*β*-acetoxy taraxer-14-en-28-oic acid, trachyloban-19-oic acid, trachyloban-18-oic acid, neoclerodan-5,10-en-19,6*β*;20,12-diolide, 3*α*,19-dihydroxytrachylobane, and 3*α*,18,19-trihydroxytrachylobane [8].

Several hydroalcoholic* C. macrostachyus* stem bark extracts have been tested against a clinical strain of* Neisseria gonorrhoeae *[9] with the minimum inhibitory concentration (MIC) of 125–250 mg/mL. There are both negative and positive reports on the antibacterial activity of methanol extracts from* C. macrostachyus* leaves [5, 10]. Contrasting results could be attributed to the locality of plant species, parts used, time of collection, storage conditions, and methods of analysis [11]. In the present study we tested the antimicrobial activity of methanol, ethyl acetate, and isobutanol extracts from* C. macrostachyus* stem bark against human pathogenic* E. coli*,* Salmonella typhi*,* Klebsiella pneumoniae*,* Enterobacter aerogenes*,* Listeria monocytogenes, *and* Candida albicans. *The ethyl acetate extract was submitted for further purification using column chromatography fractionation and the purified compound was identified by NMR spectroscopy. Agar well diffusion method [12] was used to determine the zones of inhibition and the MIC values.

## 2. Materials and Methods

### 2.1. Plant Collection and Extraction

*C. macrostachyus* stem bark was collected from Baraton Community in Nandi District of Kenya from the nature preserve of the University of Eastern Africa, Baraton, Kenya. Sample of plant was deposited in the herbarium at the Department of Biological Sciences, University of Eastern Africa, Baraton, and the National Museums of Kenya for identification.

### 2.2. Preparation of the Bark Extract

The fresh stem bark was cut into small pieces using a pen knife. The cut bark was air-dried in a shaded area for three weeks. The air-dried bark was powdered using a mechanized hand grinder. The powdered material (500 g) was soaked into absolute methanol, ethyl acetate, or isobutanol for 24 hours. The soaked extract was separated from the plant residue using a Buchner funnel. The extract was separated from the solvent by a rotary evaporator (Rotavapor R300) below 40°C. Thin layer chromatography was used to detect the presence of polar compounds. In order to isolate pure compounds from the ethyl acetate extract silica gel column chromatography was employed. The column was washed with hexane and toluene and the sample eluted and collected with ethyl acetate. The eluted samples were dried in air and crystallized. The crystals were analyzed by NMR spectroscopy.

Each plant extract was then dissolved in dimethyl sulfoxide (DMSO, Rankem, India) to the concentration of 500 mg/mL to determine the zones of inhibition. For the minimum inhibitory concentration measurements the stock solution (500 mg/mL) was serially diluted to give the concentrations of 250 mg/mL, 125 mg/mL, 62.5 mg/mL, 31.25 mg/mL, 15.63 mg/mL, 7.81 mg/mL, 3.90 mg/mL, and 1.95 mg/mL.

### 2.3. NMR Analysis

The crystallized ethyl acetate extract was dissolved in deuterated chloroform (CDCl_3_) and transferred into a 5 mm NMR tube. Subsequent NMR analysis was carried out at RT on Bruker Avance AV400 spectrometer with 5 mm Automatic Tuning and Matching (ATM) Multinuclear Broadband Observe (BBO) probe including ^1^H and ^13^C experiments. All experiments were referenced to the solvent peak.

### 2.4. Bacterial Strains and Culture Conditions

The following bacterial strains were used for the study:* Escherichia coli* ATCC 25922,* Salmonella typhi* ATCC 2202,* Klebsiella pneumoniae* ATCC 1583380,* Enterobacter aerogenes* MTCC 2990,* Listeria monocytogenes *ATCCEK 138, and* Candida albicans* ATCC 14053. The microbial freeze-dried strains were stored in the deposit of the University of Baraton and regenerated in brain heart infusion broth (HiMedia Laboratories Pvt., Ltd., Mumbai, India). The organisms were then cultured overnight on Müeller-Hinton agar (HiMedia Laboratories Pvt., Ltd., Mumbai, India). For the antimicrobial experiments one colony of each microbial strain was inoculated from the agar into 5 mL of tryptone soya broth (TSB) (HiMedia Laboratories Pvt., Ltd., Mumbai, India). The microbial strains were cultured overnight, bacterial strains at 37°C, and* C. albicans* at 30°C. The cultures were followed by measuring the absorbance at *A*_625 nm_. For the antimicrobial experiment the densities of the cultures were adjusted to the *A*_625 nm_ value of 0.1 against a TSB control using a spectrophotometer.

### 2.5. Agar Well Diffusion Method

Antimicrobial activity of the extracts was analyzed using agar well diffusion assay according to the technique described by Taye et al. [12]. Several plates of Müeller-Hinton agar were made and labeled according to the extract and by the concentration, including a negative DMSO control and the following positive antibiotic controls: for* S. typhi, E. coli, K. pneumoniae, *and* E. aerogenes *antibiotics used were amoxicillin and ciprofloxacin. For* L. monocytogenes* antibiotic controls were ampicillin and benzylpenicillin and for* C. albicans *antibiotic controls were clotrimazole and cefotaxime. The selection of antibiotics and antifungal agents was based on the general knowledge of the typical susceptibilities of the chosen indicator microorganisms, and the expected sensitivities were confirmed during the actual experiments.

Then 0.6% soft agar (Bacto Agar, HiMedia Laboratories Pvt., Ltd., Mumbai, India) (2 mL) was introduced into tubes and sterilized by autoclaving. After cooling the soft agar tubes were placed in a water bath at 48°C. 200 *μ*L of organism was mixed with 2 mL of the soft agar and the mixture was aseptically transferred to the surface of the solid Müeller-Hinton agar plate and allowed to solidify for 40 minutes. In addition to control wells three sample wells were drilled in the extract concentration-labeled agar plate using a sterilized 6 mm cork borer, giving 3 replicates per plate. The extract (75 *μ*L) was transferred to each of the 3 wells per plate. The zones of inhibition (mm) were recorded from measurements of the clear zones around the agar wells. The minimum inhibitory concentrations were determined using the tube dilution method. Results for antimicrobial tests were reported as means ± standard deviation (SD). Minimal inhibitory concentration was the lowest concentration showing clear zone of inhibition. For each solvent extract and each organism, three different experiments were conducted, giving nine readings per extract for each organism. Positive antibiotic controls were included in the assays at their recommended effective concentrations.

## 3. Results

### 3.1. Growth Inhibition

Results of the growth inhibition experiments are shown in Table 1. The ethyl acetate extract induced the zone of inhibition between 10.1 ± 0.6 mm and 16.0 ± 1.2 mm against* S. typhi*,* E. coli*,* K. pneumoniae*,* E. aerogenes,* and* L. monocytogenes* with slight antimicrobial activity against* C. albicans *(zone of inhibition: 5.6 ± 1.0 mm). The isobutanol extract showed activity against* S. typhi*,* E. coli, K. pneumoniae, *and* E. aerogenes *(zone of inhibition between 8.3 ± 1.5 mm and 13.7 ± 1.4 mm) but did not show any activity against* L. monocytogenes *and* C. albicans.* The methanol extract was active against* E. coli, K. pneumoniae, E. aerogenes, *and* C. albicans* with the zone of inhibition between 9.0 ± 1.1 mm and 14.9 ± 1.3 mm. The methanol extract was not active against* L. monocytogenes *and had very low activity against* S. typhi* (zone of inhibition: 2.3 ± 1.87 mm). All the extracts had quite equal activity against* E. aerogenes *(zone of inhibition within 10.1 ± 0.6–10.8 ± 1.2 mm). Against* S*.* typhi *and* L. monocytogenes *the ethyl acetate extract was the most active (zone of inhibition between 16.0 ± 1.2 mm and 11.7 ± 1.3 mm, resp.). Against* E. coli* the isobutanol extract possessed the highest activity (zone of inhibition: 12.2 ± 1.6 mm). Against* K. pneumoniae *and* C. albicans *the methanol extract was the most active (zones of inhibition: 14.9 ± 1.3 mm and 12.0 ± 1.4 mm, resp.). The antibiotic controls were constantly high. The DMSO negative control had no inhibitory activity.

For the active extracts MIC values were determined (Table 2). For the methanol and isobutanol extracts the MICs were 250–500 mg/mL (results not shown). The methanol extract against* E. coli *had MIC of 250 mg/mL and against* K. pneumoniae*,* E. aerogenes,* and* C. albicans* MIC was 500 mg/mL, although the methanol extract induced the narrowest zone of inhibition against* E. coli*. Isobutanol extract had MIC of 250 mg/mL against both* E. coli* and* S. typhi* and 500 mg/mL against* K. pneumoniae* and* E. aerogenes. *For the ethyl acetate extract the MICs were 125–250 mg/mL against all the studied pathogens except against* C. albicans* (MIC of 500 mg/mL). Lupeol had the lowest MIC for* K. pneumoniae* (125 mg/mL) and the highest MIC against* C. albicans* (500 mg/mL). Against* E. coli*,* S. typhi*,* E. aerogenes,* and* L. monocytogenes* MIC was 250 mg/mL (Table 2).

### 3.2. NMR Analysis

**Figure 1:**
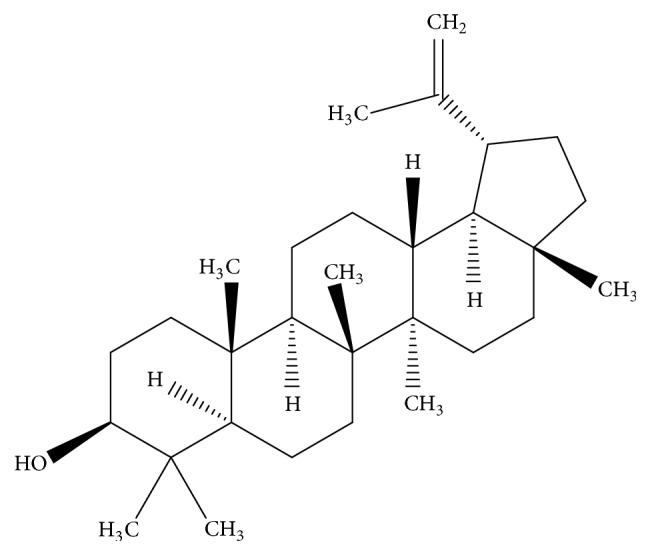


NMR analysis of the fractionated and crystallized ethyl acetate extract was carried out. The chemical shifts of the carbon atoms were compared to the literature information (Table 3) [13]. The chemical shifts showed that the purified compound was triterpene lupeol (Figure 1).

## 4. Discussion

When testing methanol extracts of* C. macrostachyus *leaves and roots Wagate and colleagues [5] found MICs between 15.6 and 250 mg/mL against three bacterial organisms,* E. coli*,* Bacillus cereus*, and* Pseudomonas aeruginosa*. In the present study the extracts from* C. macrostachyus* stem bark induced growth inhibition against all the studied pathogens including four gram-negative and one gram-positive bacteria with MIC values of 125–250 mg/mL. The results achieved in the present study are in accordance with previous results carried out by Suffredini et al. [11].

In the present study the antibiotic controls were constantly high and some extracts induced inhibition which almost reached the antibiotic level, when measured as zone of inhibition. Our results show that between the bacterial strains there is variation in susceptibility to extracts. The antimicrobial effect of the extract depends on the bacterial strain and the extraction solvent. The broadest effect against all the studied bacteria in a dose-dependent manner was achieved by the ethyl acetate extract. However, against* E. coli* the isobutanol extract and against* K. pneumoniae* and* C. albicans* the methanol extract had even higher activity. The main components of* C. macrostachyus* stem bark are lupeol, betulin, and fatty acids [6]. Because of their solubility properties one could conclude that the isobutanol and methanol extracts contained mixtures of these compounds. The active components of the isobutanol and methanol extracts remain here unknown.

Lupeol isolated from other plants has been reported to inhibit the growth of several types of bacteria, fungi, and viral species [14–20]. In the present study lupeol was isolated from* C. macrostachyus* stem bark by ethyl acetate extraction followed by silica gel column chromatography. The previous reports support our finding on antimicrobial activity of lupeol. In our study, it was the first time when lupeol was extracted from the stem bark of* C. macrostachyus* and showed broad antimicrobial activity against several important human pathogens* E. coli*,* S. typhi*,* K. pneumoniae,* and* C. albicans *with the novel finding against* E. aerogenes *and* L. monocytogenes*.

The present study gives scientific evidence for the use of the extracts and especially lupeol from* C. macrostachyus* stem bark as antimicrobial candidates against several human pathogens. They could be utilized as preventive agents in disinfectants or submitted to further process for drug or dietary supplement development. The provision of safe and effective traditional medicines could offer increasing access to health care [1]. Various studies have established that herbal medicines can be developed as safe, effective, and less costly alternatives to the current medicines to treat certain bacterial infections [21].

## Figures and Tables

**Table 1:** Zone of inhibition of the different *C. macrostachyus* extracts against selected human pathogens.

Pathogen	Zone of inhibition				
	Extract			Antibiotic	
	^1^MeOH	^2^EtOAc	^3^BuOH	A	B
*Escherichia coli*	9.0 ± 1.1	12.2 ± 1.6	13.7 ± 1.4	20.1 ± 0.9	16.2 ± 0.8
*Salmonella typhi*	2.3 ± 1.87	16.0 ± 1.2	8.3 ± 1.5	22.1 ± 0.9	20.8 ± 0.8
*Klebsiella pneumoniae*	14.9 ± 1.3	10.7 ± 1.0	11.1 ± 1.9	19.7 ± 0.5	17.3 ± 0.9
*Enterobacter aerogenes*	10.8 ± 1.1	10.1 ± 0.6	10.8 ± 1.2	21.3 ± 0.5	18.6 ± 0.5
*Listeria monocytogenes*	0.0 ± 0.0	11.7 ± 1.3	0.0 ± 0.0	21.0 ± 0.8	18.3 ± 0.5
*Candida albicans*	12.0 ± 1.4	5.6 ± 1.0	0.0 ± 0.0	21.5 ± 0.7	13.4 ± 0.7

^1^MeOH, methanol extract.

^2^EtOAc, ethyl acetate extract.

^3^BuOH, isobutanol extract.

Zone of inhibition (diameter in mm) including diameter of the well (6 mm). The experiments were carried out using triplicate samples. The results are from three independent analyses and are the mean of 9 values (*n* = 9).

In negative control DMSO no inhibition was found.

For *E. coli, S. typhi, K. pneumoniae, *and *E. aerogenes *antibiotic A was amoxicillin and antibiotic B was ciprofloxacin.

For *L. monocytogenes* antibiotic control A was ampicillin and antibiotic control B was benzylpenicillin.

For *C. albicans *antibiotic control A was clotrimazole and antibiotic control B was cefotaxime.

**Table 2:** Minimum inhibitory concentrations (MICs) (mg/mL) of *Croton macrostachyus *EtOAc extract against selected human pathogens.

Pathogen	MIC
*Escherichia coli*	250
*Klebsiella pneumoniae *	125
*Enterobacter aerogenes *	250
*Listeria monocytogenes *	250
*Candida albicans *	500

**Table 3:** NMR analysis of the ethyl acetate extract from *C. macrostachyus* stem bark^1^. Chemical shifts of the numbered carbon atoms are shown. Numbering of the carbon atoms used is that of triterpene framework.

Carbon number	Sample (ppm)	Lupeol (ppm)
28	18.22	17.97
20	151.20	150.88
29	109.54	109.31
3	79.23	78.94
17	43.22	42.95
5	55.51	55.25
9	50.65	50.38
19	48.20	47.94
18	48.51	48.24
14	43.04	42.78
8	41.04	40.78
4	39.07	38.81
1	38.92	38.67
13	38.26	38.00
22	40.20	39.96
7	34.49	34.23
16	35.80	35.54
15	27.66	27.41
21	30.06	29.80
23	28.20	27.95
2	27.62	27.35
10	37.38	37.11
12	25.35	25.08
11	21.14	20.89
30	19.52	19.28
6	18.50	18.28
26	16.19	15.94
25	16.33	16.09
24	15.59	15.35
27	14.76	14.51

^1^Obtained on a Bruker Avance AV400 spectrometer and referenced to the solvent peak (CDCl_3_ at 77.00 ppm).

## Abbreviations

DMSO: Dimethyl sulfoxide
NMR: Nuclear magnetic resonance
CDCl_3_: Deuterated chloroform
MIC: Minimal inhibitory concentration.
